# Supplementary material for: Patterns of Intron Gain and Loss in Fungi
Source: PLoS Biol. 2004 Nov 30;2(12):e422. doi: 10.1371/journal.pbio.0020422 (PMC532390; doi:10.1371/journal.pbio.0020422)
Supplement: Table S1 — Also available at http://genes.mit.edu/NielsenEtAl/. (4.3 MB ZIP). [file pbio.0020422.st001.zip › NielsenEtAl/html/980.html]

AN4186.1.NCU09035.1.MG08318.1.FG09882.1


```
 CLUSTAL W (1.82) Multiple Sequence Alignments - Introns Inserted


Sequence 1: NCU09035.1	267 aa
Sequence 2: MG08318.1	271 aa
Sequence 3: FG09882.1	272 aa
Sequence 4: AN4186.1	267 aa
Alignment Length: 285 aa
Number Identitical Residues: 133 aa
Alignment Score (without introns) 6262


MG08318.1 	-MPPTNLLAGKTAIITGGTTGIGR~SIALEFLRQGCSVAVNHLGLESDRAHLESLVAEAE
NCU09035.1	-MPPTNLLASKTAIITGGTTGIGR~AITLAFLAQGCSVAVNHLDLPRDRHHVDSLIAEAA
FG09882.1 	MLMPAGLLANKTAIITGGTTGIGR~AICLEFLRQGANVVVNHLGLEKDQTHLDSLIVEAD
AN4186.1  	-MSLPQLLNGKVAAITGGLTGIGR0AIALEYLRHGAYVAVNHLGGSNDEPLLTAMKKDVS
          	 :  . ** .*.* **** ***** :* * :* :*. *.****.   *.  : ::  :. 

MG08318.1 	AISSSS--------ATAGRLTHLPGDVREPATGTALVSHALTALSSSRLDICVSNAGICT
NCU09035.1	ALKSSSSSSSSPQHTTIGSLALLPGDVRDPSTGPALVKFALDTFKTNRLDICVSNAGICQ
FG09882.1 	EIRKAS--------PTAGHLDHQAGDVRDPATATELVKKAVEHSPKKRLDICVSNAGICT
AN4186.1  	ELLQQTS------QTDKERFITVSGDISKPETGTEFIEKTVQAFG--RLDVFVSNAGVCR
          	 : . ::       .    :   .**: .* *.. ::. ::      ***: *****:* 

MG08318.1 	FAEFLD~LDAALYDKTARTNLDGCFYVVQAAARQMARGQTPPGGSIIGVSSISALVGGGL
NCU09035.1	FADFLD~MDADLFSNTVRTNLDGAFYVVQAAARQMARHQDPPGGSIIGISSISALVGGAG
FG09882.1 	FADFLT~LEPDLLHSTVRTNLDGAFYVTQAAARQMALHQEPKGGSIIGVSSISALVGGGQ
AN4186.1  	FEEFLD2VTPQLLNHTITTNLTGAFYAVQAAARQMAFSQSPPGGSIIGISSISALVGGGQ
          	* :**  : . *   *  *** *.**..********  * * ******:*********. 

MG08318.1 	QTHYTPTKAGVLSLMQSTAVALGRYGIRCNSLLPGTVRTQLNEEDLRDDK-----KREYM
NCU09035.1	QTHYTPTKAGVLSLMQSMACALGRYGIRCNAILPGTVETQLNEGDLNADEEEGREKRRYM
FG09882.1 	QTHYTPTKAGVLSLMQSTACALGEHGIRCNALLPGTIRTQLNDADLADDT-----KRAYM
AN4186.1  	QTHYTPTKAGVLSLMQSTAVALGKYGIRCNALLPGTIRTQLNDEDMSDPV-----KRKYM
          	***************** * ***.:*****::****:.****: *:         ** **

MG08318.1 	EGRIPLGRTGEPKDLAGPAVFLACEELSG~YV~TGAQLLVDGGLFVNLQ
NCU09035.1	EGRIPLGRLGQPKDVGGPAVFLACEELSG2D-~----------------
FG09882.1 	EGRIPLGRTGSPSDMAGPAVFLACPELSG~YV~TGAQLLVDGGLFVNLQ
AN4186.1  	EGRIPLGRLGQPPDLAGPAVFLACEGLSG~YV0----ILVDGGLFVNLQ
          	******** *.* *:.********  ***           ...   . .
```
